# Modbus Extension Server Implementation for BIoT-Enabled Smart Switch Embedded System Device

**DOI:** 10.3390/s24020475

**Published:** 2024-01-12

**Authors:** Vasile Gheorghiță Găitan, Ionel Zagan

**Affiliations:** 1 Faculty of Electrical Engineering and Computer Science, Stefan cel Mare University, 720229 Suceava, Romania; 2 Integrated Center for Research, Development and Innovation in Advanced Materials, Nanotechnologies, and Distributed Systems for Fabrication and Control (MANSiD), Stefan cel Mare University, 720229 Suceava, Romania

**Keywords:** communication, Modbus extension, acquisition cycle, server, Building Internet of Things, remote terminal unit (RTU)

## Abstract

The industrial control and automation sector has invested in the development and standardization of new wireless (WirelessHART, ISA 100.11a, and WIA-PA) and wired (Profibus/Profinet, Modbus, and LonWORK) solutions aimed at automating processes to support standard monitoring and control functions from the perspective of addressing critical applications, as well as those integrated within the Building Internet of Things (BIoT) concept. Distributed data acquisition and control systems allow modern installations to monitor and control devices remotely. Various network protocols have been proposed to specify communication formats between a client/gateway and server devices, with Modbus being an example that has been widely implemented in the latest industrial electrical installations. The main contribution made in this paper concerns the completion of the Modbus Extension (ModbusE) specifications for the server station in the classical Modbus communication architecture, as well as their implementation and testing in an STM32F4 kit. A general-purpose control architecture is proposed for BIoT sector, comprising both intelligent touch switches and communication protocols of which the Modbus protocol is used extensively for the monitoring and control part, especially between clients, smart switches, and devices. The specific contributions concern the presentation of a scientific and practical implementation of improved specifications and their integration as software modules on ModbusE protocol server stations. A client station with a VirtualComm USB PC connection is also implemented in the lab to test the operation of the proposed server with specific Modbus applications.

## 1. Introduction

Today, interest in Smart Cities (SCs), Smart Buildings (SBs), and Smart Homes (SHs) is growing due to the particular technological developments driven by the rapid development of the IoT, wired and wireless communication networks, the extensions of communication protocols such as Modbus [1,2,3], touch screen technologies, LCD, microcontroller performance [4], and the use of cloud computing (CC) and smartphones. Academia, industry, government, society, and the natural environment provide support for the development and application of new technologies, while the active involvement of citizens can be considered crucial for this strategy; therefore, it is clear that the work and involvement of scientific researchers are part of SH solutions [5].

Building automation includes a network of hardware and software interfaces and electronic circuits. It integrates various appliances and devices around the building via the Internet [6,7,8]. The communication of these devices within larger system networks, e.g., with the Internet, can be achieved via wired communications such as RS485 with or without proprietary protocols (Modbus, KNX, BacNET, LonWORK, M-Bus or Meter-Bus, other local industrial networks such as CAN, CAN-FD, XL, Profibus/Profinet, or Ethercat, or wireless ones such as Wi-Fi [9,10], Bluetooth [11,12], ZigBee, LoRaWAN, SigFox, and 6LoWPAN). With the exception of Wi-Fi, the other networks need a gateway or controller with a connection to the local network or the Internet.

The important technological advances that were presented for SHs are related to the development of Industry 4.0, which symbolizes the beginning of the fourth industrial revolution and represents the current trend of automation technologies in industry. This mainly includes enabled technologies such as Cyber-Physical Systems (CPSs), IoT, and CC, which integrate virtual space with the physical world. Before the advent of these technologies, installing and configuring an SH system was a complicated step, as wired systems and infrared sensors were used, which were considered challenging, complicating the installation step and affecting user comfort. Nowadays, wireless technologies for SHs, such as Wi-Fi, Bluetooth Low Energy, and ZigBee [13,14], are helping the development of SH systems and SCs [15], in addition to enabling remote control via smartphone apps [16,17]. Classical SHs, IoT, and CC are the building blocks for achieving an advanced level of integrated home automation systems. Each component brings its own attributes and core technologies [18,19,20,21]. The specific problems that needed to be solved concerned the synchronization of spatially distributed measurement devices and the collection of data from multiple devices in large-scale experimental setups. The Modbus protocol extension is based on these new technologies introduced at the UART level. The proposed extension retains all the features of the Modbus RTU protocol except for the bit structure of a character. To support this, we can have advantages given by situations generated by bus access (these cannot occur because the dominant bit always wins and the driver circuitry will not be stressed), and the three resistors used by the RS485 line driver to set the line insensitivity zone, usually used at one end of the line, can be eliminated, driver direction monitoring is not required, or whether or not the transmitted data are identical to the received data can be checked.

Compared with the previous publication [22], the server module proposed in this paper is a novelty because it is the first ModbusE (MBE)-based implementation integrated into an SH. The system required the implementation of the Modbus RTU protocol at BIoT (smart switch) server stations, respectively, as well as the completion and definition of new requirements for the Modbus protocol extension called ModbusE at the MBE_RTU server station level with 100% compatibility with the maintained Modbus RTU protocol [23]. The STM32F429I-DISCO microcontroller development kit, the STM IDE Cube application development tool, and the FreeRTOS operating system were used for laboratory modeling and experimental testing, presenting an example of ModbusE and IoT-based SH usage. The ModbusE architecture is based on the ISO/OSI 3-level reference model, i.e., levels 1, 2, and 7. The performance improvement is based on new microcontroller technologies and better data rates with increased communication speed, character length, and embedded applications. 

The contributions made in this paper concern the server implementation included in the BIoT and SHs that is based on the MBE protocol extension; this is intended to extend the classical Modbus specification with the following purposes:

- To transform the Modbus protocol into a fully defined protocol by specifying a flexible acquisition cycle and a BIoT device description language;
- The current implementation as a derived contribution contributes to achieving deterministic behavior by implementing the acquisition cycle and assignment of tasks to ensure temporal consistency of information when certain decisions need to be made: decisions based on a set of data produced by distributed systems and applications.
- An algorithm is proposed for StartTaskServerMBECycle task implementation logic dedicated to serverMBE stations.

The following goals are defined for ModbusE or the acquisition cycle of gateMBEx:

- The acquisition cycle can have *n* slots, where *n* ϵ 3 ÷ 246, with each slot supporting *n*_0_, *n*_1_, …, *n*_n−1_ characters sent and received;
- The minimum number of slots in an acquisition cycle is 3;
- A slot consists of *n*_i_ × 10 × θ ticks;
- Each slot has a data structure attached with status, control, and data;
- A ModbusE station, by configuration, can subscribe to multiple transmit/receive slots.

The remainder of this paper is organized as follows: Section 2 reviews current BIoT- and Modbus-based projects. Section 3 describes the conceptual model for the proposed server version based on the acquisition cycle of ModbusE communication, and Section 4 validates the implemented laboratory model based on the MBE server. Section 5 presents the conclusions and future research.

## 2. Related Works

In the paper in [24], the authors conduct research on the unreliability of the Modbus protocol. Thus, a scenario of injecting false commands hidden by the SCADA controller is realized. Such a scenario would cause significant damage to SCADA systems. Thus, following the research carried out, the authors propose valuable solutions to eliminate these situations. The attack scheme integrates two main phases. The first preattack phase is carried out in offline mode, namely, an attacker sniffs, collects a sufficient number of pairs of valid response requests, and stores them in a database. After this initial stage, the attack phase in online mode is presented. In this stage, the attacker sends the false command and retrieves a valid response from their database, and they do so for every request sent by the HMI user. In the paper in [25], a new address translation scheme is proposed through which the slave PLCs use the serial Modbus protocol in a SCADA system which gives the possibility of integration with IEEE 802.15.4. Thus, XBee modules do not require a specific PLC configuration. At the same time, the scheme proposed by the authors effectively eliminates the need to link any XBee module to a specific PLC. In this way, the paper describes the hardware architecture on which the scheme proposed by the authors is implemented. The process of realizing address translation and Modbus integration with the IEEE 802.15.4 network is also presented. As a contribution from the authors, the address translation scheme can be highlighted.

The paper in [26] proposes a method for designing a measurement system using the Modbus TCP. The authors successfully present the structure of the measurement system and a description of the algorithmic software that implements the telecommunication function. In [27], the authors conduct research on monitoring PLC energy meter data using Modbus over RS485 through an application-based monitoring system to detect irregularities that occur remotely. In the proposed implementation, if there is any anomaly on the distribution line on the HMI, an alert is displayed indicating that the set parameters have been deviated and, in addition, the Node-Red platform will keep track of the data. Therefore, the system proposed by the authors was tested and obtained the desired results in the context that there are several specialized automation protocols and standards that can be used. 

In the paper in [28], a Modbus RTU/TCP to Profibus gateway is designed. This attempts to solve the incompatibility problem between Modbus and Profibus. Raspberry Pi manages the functionality of the complete system and handles the Modbus traffic communication, while VPC3 + S controls the Profibus-DP communication layer. Experimental results obtained by the authors based on Modbus and Profibus communication demonstrate the correct operation of the dedicated gateway consisting of a Raspberry Pi and the VPC3 + S intelligent chip. In [29], the authors design and implement an RTU server for verification intellectual property (VIP) development and testing that has been intentionally subjected to errors to test the VIP. Simulations were performed using Questasim 2021.4. The VIP capability was able to trace all errors in the embedded design as a fraction of the error sequence. An alternative Modbus control design was successfully simulated with the VIP where the server was also able to identify errors in the form of protocol faults.

The paper in [30] presents an educational guide for integrating the EcoStruxure Power Monitoring Expert application with Schneider Easergy P3/P5 protection relays as well as the IEC 61850 communication standard [31]. The contribution of the authors concerns proper and effective teaching, i.e., the use and application of these tools in industrial protection monitoring. The work helps to strengthen training in key areas for the industrial sector and promotes practical and applied training of great importance for the training of engineers and technicians in the field of industrial automation and communication. According to [32], in the midst of various CPS entities, AMI (Advanced Metering Infrastructure), i.e., the smart grid, is among the most important entities essential for the rapid transformation of CPS, where Modbus protocol (RS-485) is commonly used in smart meters for communication on the physical layer. The Modbus Attack DataSet for AMI (MAMI) is experimented on, and the authors’ results demonstrate that the FL design approach is remarkably efficient in identifying critical smart meter assaults. In addition, data confidentiality concerns are protected. 

In [33], the use of the Modbus protocol for SB security is presented. Thus, an embedded network communication protocol gateway has been designed that bridges the traditional serial Modbus fieldbus interface and Ethernet connectivity, and the implementation is built on the Modbus TCP interface. The authors propose a Modbus-protocol-based design for SB security so that the proposed system adopts the Modbus protocol format for data. Also, an ARM5708-based hardware system as the development and design support of the development board, Linux OS, JavaWeb, and database management technology realizes the data interaction, storage, display, and identification. To meet the demand of embedded data acquisition monitoring systems of industrial automation applications, a Modbus- and Linux-protocol-based platform is proposed and extensively presented in the paper in [34]. Thus, the Modbus client realized by this embedded platform is stable and reliable. It also has excellent prospects in data acquisition monitoring systems incorporated in new automation applications. As a result, communication with various serial equipment Modbus protocol can be successfully tested by implementing Modbus for a serial port, which includes two types of communication modes, namely ASCII and RTU. 

## 3. Basic Architecture of serverMBU_i Application with ModbusE

**Figure 1:**
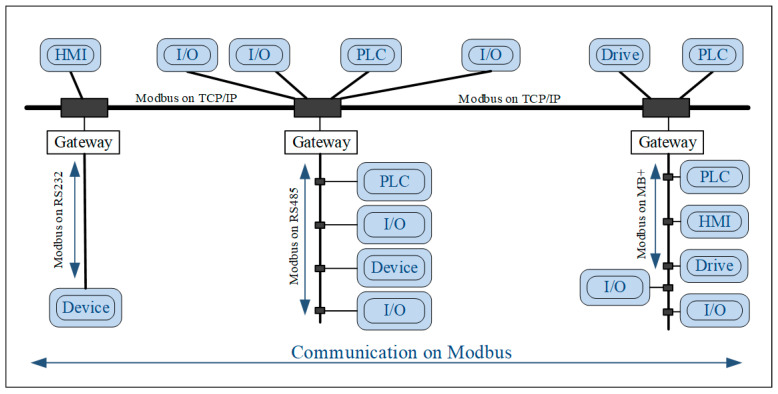
Modbus network example.

The success of Modbus comes from the compromise to keeping it simple, while recognizing that factory automation applications are not and that there are also benefits in delegating the management of application diversity [35]. An example of a Modbus network is illustrated in Figure 1. As you can see, gateways are employed to interconnect the lower layers of the network, HMIs represent human–machine interface terminals, and PLCs constitute programmable logic controllers. Modbus messages/transactions are used to exchange data units for application protocols [36].

While the Application Protocol Data Unit (APDU) is identical for all Modbus lower layers, the client/server scheme used by Modbus exploits the full potential of the different possibilities afforded by the lower layer presently in use. The following is a summary of the ModbusE extension described in the literature in [2,22]. Thus, ModbusE uses two types of objects:

- PDO (Process Data Object) for transferring processing data;
- SDO (Service Data Object) for setup, servicing, and testing.

The communication of these objects over the network is carried out with messages, with several messages making up transactions. There can be three types of messages:

- SDA (Send Data with Acknowledge) sends data with acknowledgment (request–response);
- SDN (Send Data with No Acknowledge) sends data without acknowledgment (request);
- SRD (Send and Request Data) sends and requests data (request–response).

From the point of view of the communication paradigm, the following architectures can be distinguished:

- Master–Slave (Client–Server);
- Producer–Consumer.

ModbusE maintains compatibility with Modbus RTU. Table 1 shows the set of tables that feature distinct functionalities. The differences between inputs and outputs and bit addressable versus addressable at the 16-bit word level of the data items do not involve any software functionality. It is completely permissible and highly common to deem all four tables as overlapping, if this is the very natural representation of the specific target host. For individual primary tables, the protocol enables a single selection of 65,536 data entries, and read or write operations on those entries are intended to scale across multiple sequential data items up to a data size limit that relies on the operation function code. It is evident that all data managed by Modbus, such as bits or registers, needs to be located in application memory on the machine. However, the physical address in memory may not be mixed up with the reference data. The only condition is to link the reference data with the physical address. Modbus logical reference numbers, which are commonly referred to in Modbus functions, are unsigned integer indices beginning at zero [36]. 

The application level for Modbus precisely specifies the addressing logic of the PDU. In each Modbus PDU, every datum is addressable from 0 to 65,535. Furthermore, it precisely describes a Modbus data model consisting of four blocks with multiple elements ranging from one to n. Within the Modbus data model, the elements in a data block are individually referenced from one to n. 

Subsequently, the Modbus data model must be linked to the device application, IEC-61131 object, or other application model. The preliminary mapping between the Modbus data model and the device application is entirely vendor-device-specific. In terms of the operating model of the server, i.e., the smart switch device, a holding register with a specific address will store a specific value and a different number of buttons will be displayed on the touch interface depending on this value. Another ModbusE address can be assigned to configure the effects of touching the buttons so that button functionality can be selected in two modes, namely toggle or nontoggled state. In the same mode, the audible mode of operation can also be implemented, whereby each button can also have audible effects. This operation, once activated, will be valid for all buttons, and a specific ModbusE register will manage the sound effect for each button.

### 3.1. ModbusE Acquisition Cycle Structure

**Figure 2:**
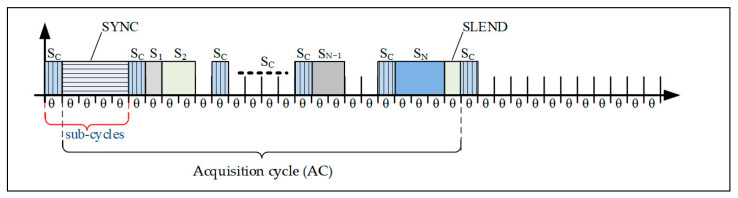
A possible solution for a control loop acquisition period shorter than the AC [23].

**Figure 3:**
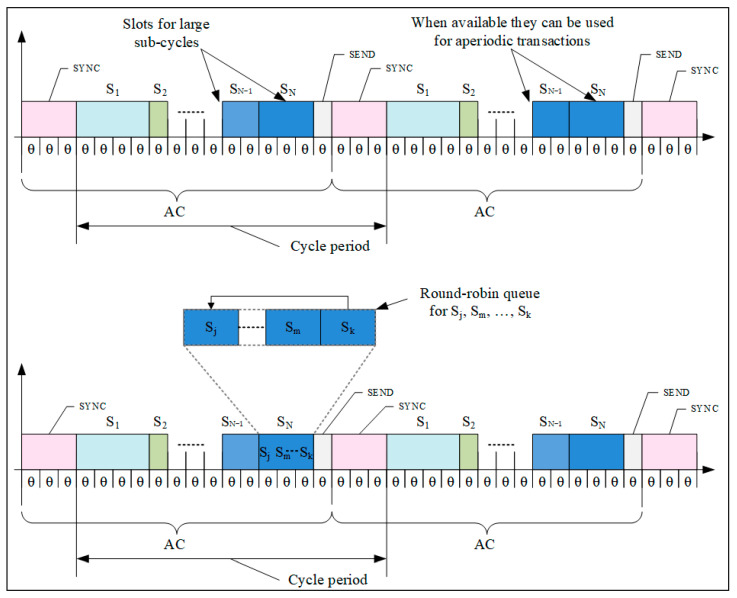
ModbusE uses slots within the acquisition cycles or aperiodic transaction slots for slots with large subcycles and slots for aperiodic transactions (the SN slot must be larger than any slot in the round-robin queue, and subcycle slots have higher priority than aperiodic transaction slots.

In a ModbusE acquisition cycle, as shown in Figure 2 and Figure 3, the following statements are summarized:

- The basic time unit is referred to as tick (θ). The size of this tick is chosen so that it is supported by all stations in the local industrial network.
- The acquisition cycle (AC) consists of time slots (S) of length l (i.e., lθ). It follows that slots can have different lengths. These are chosen at setup and, during operation, are considered fixed slot lengths.
- Slots may have priority (PRi).
- Within a cycle there are periodic and aperiodic slots. At least one slot must be aperiodic for SDO communication. It must be long enough to allow the whole transaction to take place.
- The slot allocated to SDO messages should not exceed 10 ÷ 15% of the value of the acquisition cycle.
- A particular case is where slots, PDOs and SDOs, are being sent based on separate queues. PDOs are placed in a queue and are sent based on a round-robin algorithm, and SDOs are placed in a FIFO queue. If there are SDOs in the queue, allocate an alternative communication medium, i.e., a PDO or an SDO.
- Within the acquisition cycle there can be subcycles (SACs). The network collects messages characterized by type (SDA, SDN, or SRD) and maximum transfer Mi(type, t). Thus, the message is indivisible.
- A transaction is composed of several TRi(t) messages and is indivisible.
- A transaction or message occupies one slot in the cycle.
- An erroneous message or communication error is not allowed to delay the slot. However, for unpredictable situations, the cycle will extend with a safety interval.
- The cycle can also be provided with an alarm or emergency slot and possibly with a slot (the first one) for synchronization.

The acquisition cycle consists of a slot called SYNC (optional) that can be used by the client (gateMBE station as Modbus RTU client) to initiate a broadcast command, such as “start scanning inputs” or “a new acquisition cycle has started”.

The cycle optionally ends with a SEND (Slot END) end-of-cycle slot, which indicates the end of the acquisition cycle and may possibly retrieve some errors that led to the lengthening of some of the slots. Slots S1 to SN allow for transactions whose length is a multiple of a tick (θ). Optionally, the tick can be omitted by expressing the slot directly, for example, in microseconds. For simplicity, the option of using equal slots can also be chosen. The acquisition cycle time (tAC) is simply calculated with Equation (1).

tAC = tSYNC + tSEND + tS1 + ……. + tSN(1)

### 3.2. User Modbus Commands Handled by ModbusE

At least the following new commands are required to implement the ModbusE protocol:

- PDO transmit/receive mapping command;
- Command to read a transmission/reception for PDO;
- Command mapping of physical addresses, slot addresses, and classic Modbus commands.

In the Modbus protocol, functions between 100 and 110 are public. We propose to use codes 100, 101, and 102 for these three types of functions. Mapping allows Modbus messages to be sent without a header, so overhead can be reduced. The primary benefit of having timestamps added to a simple protocol such as Modbus is time consistency, i.e., a single vision of the managed and controlled process. Time consistency becomes particularly relevant in the deployment of industrial process control algorithms. By adding this extra information, we can introduce a low-cost characteristic specific to advanced protocols. This option is implemented at the fieldbus level for Modbus, as the timestamp is added by the BSG, both on the controller and on the ModbusE fieldbus. These options are either ticked or not at the set-up phase of the BSG and in terminals that have ModbusE capability. The ModbusE 100 function, map transmit/receive PDO, is a classic Modbus message for mapping so that the headers for the request are sequential and do not exceed the number of bytes in the function header. The ModbusE function 101 reads the mapping for PDO transmit/receive PDO. The total number of bytes of the message must be fewer than 253. If one of the component messages is unknown to the server station, the mapping is not executed and the corresponding error code is returned. The ModbusE 102 function configures the server stations so that the request contains the new address for the SDO (1 byte 129 ÷ 249).

### 3.3. BIoT System Architecture Using gateMBEx 

**Figure 4:**
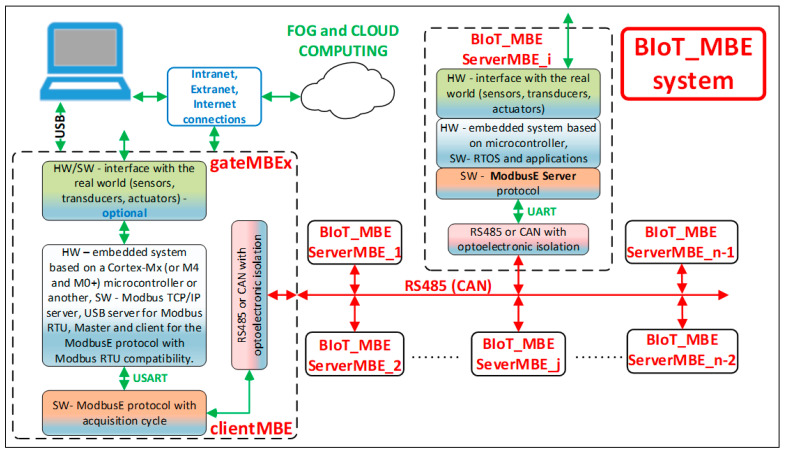
BIoT and IIoT system architecture using gateMBEx.

Figure 4 shows how the gateway fits into a more general BIoT or even Industrial Internet of Things (IIoT) system. In this sense, gateMBEx must provide the following:

- A Modbus TCP-IP server with an Ethernet connection and optional Wi-Fi;
- A Modbus RTU server with VirtualComm connection using the USB port, optional Bluetooth communication, GSM, etc.;
- The two servers will also execute local Modbus commands that will allow access to the hardware and software resources of the gateway, including all the data handled by the ModbusE protocol. In this way, the application on a host system, a client of these servers, can use classic drivers and applications specific to the Modbus protocol. Thus, it is not necessary to write a specific ModbusE driver; only the data will be defined and interpreted accordingly.
- A ModbusE server will manage the protocol extension as a client and will service the requests of Modbus TCP-IP and RTU servers. External devices do not have direct access to this server, but they do via Modbus servers.

The ModbusE server must map application objects to readable and writable Modbus objects in order to set or retrieve application object attributes and provide a way to initiate services at the application object level. At runtime, the Modbus server must analyze the received Modbus request, process the requested action, and send back a Modbus response. Modbus record and tab data types are less well-known, being used with only a few function codes. The Modbus record data type, from the point of view of an application user (client), is a set of registers, characterized by the address of the first register and their number. In the context of this definition, the registers involved have also been called references. Server stations implementing the ModbusE protocol will benefit from all its advantages, while those implementing Modbus RTU, if they support communication speed, will work in normal Modbus RTU mode.

If gateMBEx is connected to a system running OPC UA applications implementing AMQP (Advanced Message Queuing Protocol) and supporting Modbus RTU and TCP-IP drivers, then gateMBEx can be easily integrated into an IIoT architecture like the one shown in Figure 4. 

A higher degree of integration can be achieved using the new multicore microcontrollers in Cortex-Ax and Cortex-Mx combinations or other specific architecture by implementing the OPC UA server on the gateMBEx itself (gate_MBEx_UA). Thus, the communication speed is 10.5 Mbit/s with 30 slots. In the ModbusE-based server implementation, in addition to the myTaskCycle.c acquisition cycle implementation task, the following tasks were ported and tested:

- myTaskUsbServer.c: Implements Modbus RTU on VirtualComm using the USB port. The USB server sends the request to the USB client.
- myTaskMbeGate.c: It has a limited role in the USB communication being provided for Modbus TCP/IP, and USB-TCP/IP is used for compatibility at the socket.
- myTaskDispathS.c: It takes the command from the USB client and sends it to the MBE dispatcher server, which processes it and sends it to the MBE client that executes it, and the response is then sent back to the dispatcher server, which sends it back to the USB client, and from there, it goes to the USB server, which then sends it to the USB client, e.g., a PC application.
- AppMBEglobatData: It is a file filled with global data to be accessed as Modbus and MBE registers.

## 4. Proposed Modbus Extension Server Implementation

### 4.1. ServerMBE_i Station Requirements

This section presents the most important requirements for MBE servers. Some requirements are also added throughout this paper. The communication requirements for MBE servers, including ModbusE, are as follows:

- Support the communication speed and structure of ModbusE messages and frames;
- Determine correctly and efficiently the end of a message (minimum 3.5 characters);
- There must be a known timeout from receiving a message to replying to it, if applicable (can only be unanswered subscription);
- Receive all slot messages in the acquisition cycle, or at least the following:
  a. Slots 0, 1 (if 1 is included in the acquisition cycle) and asynchronous slot;
  b. Slots to which it responds (publishes) and where it is the only one that responds to the client (request–response);
  c. Slots that subscribed to either on request or response but are not the server responding to the request. In this case, serverlMBE_i may be the client (subscriber) of the gateway or another serverMBE0j. This also implements publisher/subscriber communication, with message delivery provided in hardware by the RS485 multipoint line bus.
- Be programmable for certain MBE facilities either OFFLINE, ONLINE, or preferably both;
- Have a procedure for removing and reinserting the serverMBE_i station from and into the system;
- Be able to be reprogrammed online without changing the acquisition cycle structure;
- Know and execute those commands (from the 100 series such as 100, 101, 102, …) for slot operations;
- Have a wrapper that converts the data in the data area of an MBE message into one or more classic Modbus messages to maintain compatibility with eventual software using classic Modbus messages. This would make it easier to upgrade some Modbus applications to ModbusE applications;
- Create a language to describe MBU server stations.

**Figure 5:**
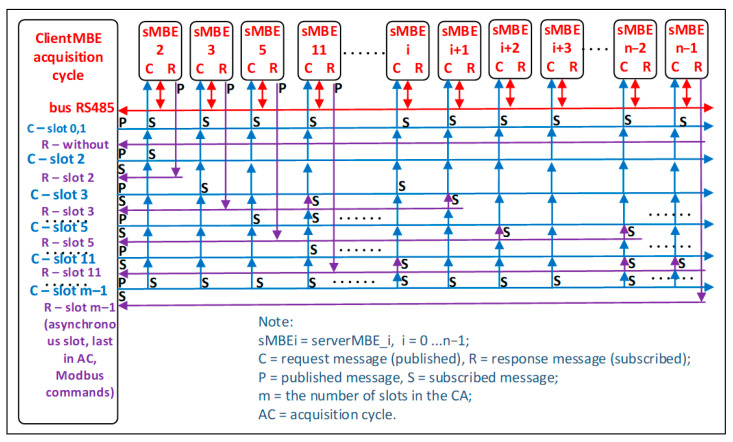
Connection examples for ModbusE.

Figure 5 shows some examples of connections for ModbusE. Thus, for slots 0 and 1, if the presence of slot 1 is set in the AC, a request (publication) is issued by the MBE client on the gateway (publication to which all stations must subscribe). For slot 2, a request (publication) is issued by the gateway MBE client to which only serverMBE_2 (hereafter referred to as sMBE2) subscribes, and it may publish a reply message to the gateway client to which the gateway has a mandatory subscription, i.e., it is checked whether the server has received the message correctly. For slot 3, a request (publication) is issued by the MBE client on the gateway to which sMBE3 and sMBEi also subscribe. sMBE3 thus publishes a reply to the client on the gateway, and the gateway, sMBE11, and sMBEi + 1 subscribe to this reply (publication) of the sMBE3 server. For slot 5, a request (publication) is issued by the MBE client on the gateway to which sMBE5 and sMBE11 subscribe, and sMBE5 publishes a response to the client on the gateway. The gateway, sMBEi-2, and sMBEn-2 subscribe to this response (publication) from the sMBE5 server. For slot 11, a request (publication) is issued by the gateway MBE client to which sMBE11 subscribes, and sMBE11 publishes a response to the gateway client. Subsequently, the gateway, sMBEi, sMBEin-1, and sMBEn-2 subscribe to this response (publication) from the sMBE11 server. For slot m-1, the last slot in the AC, a request (publication) is issued by the gateway MBE client to which sMBEn-1 subscribes, and sMBEn-1 publishes a response to the gateway client. Only the gateway subscribes to this response (publication) of the sMBEn-2 server, since it is a typical Modbus command to which only the station having the same address as the (slot) number sent by the last slot in the cycle can respond. To eliminate confusion, these classic Modbus addresses must be greater than or equal to 0x80. As an observation, it should be noted that slots in or out of the cycle (<0x0c80) may also contain Modbus messages in classic format. 

The sMBE2, 3, 5, 11, n−1 are those that publish in response to a request (publish) from the MBE client for slots with the same ID number on the RS485 network, but this is not mandatory. Servers sMBEi, i + 1, i + 2, i + 3, and sMBEn-2 only subscribe to some MBE client requests (publications) from the gateway or to some responses from other servers. As a result, like the gateway, these stations are MBE clients and eventual servers on the asynchronous slot. The architecture also considers publisher–subscriber communication.

### 4.2. ModbusE Implementation in the STM32F4 Microcontroller

STM32F4 communication devices have a single transmit buffer and a single receive buffer [37]. As a result, user software must read data from the receive buffer before the data are overwritten by the next received data. By using interrupts, there is a risk that the data will be overwritten. Furthermore, if each received character generates an interrupt, the microcontroller will be requested for additional processing time, and the time consumed with communication may become significant. The DMA in the STM32F4 prevents data overwriting, but usually, the number of bytes of data to be received is not known. For the ModbusE protocol, the byte count is usually known after the reception of four characters. As a result, the receiving end of the transfer is known later. The solution is to implement an emulation of a FIFO based on DMA and interrupts. A timeout is required for the DMA in order to indicate to the application that no further data will be received. Thus, the DMA facilities in the STM32 microcontrollers significantly simplify FIFO implementation. The DMA facilities that simplify FIFO implementation are the following:

- Transfers independent of source and destination size (byte, half-word, word) to emulate data packing and unpacking;
- Support for circular buffer management;
- Access to flash memory, SRAM, and APB1, APB2, and AHB peripherals as source and destination;
- Programmable number of data that can be transferred (up to 65,536).

All of these facilities work together to minimize the software overhead associated with data storage. The DMA’s memory address incrementing mode is very useful because the data pointer can be incremented automatically. Thus, the DMA buffer emulates the FIFO buffer, the write buffer pointer (DMA pointer) is automatically incremented, and the DMA counter is automatically decremented when writing to the FIFO. The read buffer location is incremented by software each time data are retrieved from the FIFO buffer. When FIFO is implemented by software, the data are received and stored in a circular DMA buffer, where they remain until they are managed and removed. The transmitter sends n data items using DMA, and the message length n is known in advance. The receiver receives m data items using DMA, a potentially unknown number, but it is still possible to pick up the RXNE interrupt from the peripheral, even when using DMA. In fact, the interrupt from the peripheral emulates the “buffer is not empty” FIFO interrupt. The following considerations can be made for reception:

- There is no need to clear the RXNE flag in the interrupt handling routine as it is automatically cleared by the DMA read operation. However, the interrupt remains captured in the NVIC even if the RXNE flag is no longer set;
- Two buffers are used, namely RxBuffer2 and RxBuffer2_SW. The RxBuffer2 buffer emulates the FIFO buffer and is defined as the DMA base memory address from which the data will be read. RxBuffer2_SW is a software buffer used within the interrupt routine on receive to transfer data from the FIFO. It is the final destination of the stored data.
- Within the interrupt handling routine, the DMA address/number pointer is used to indicate how many bytes of data are available in the FIFO buffer (RxBuffer2) to be transferred to the final storage buffer (RxBuffer2_SW) and what the current FIFO location of the data is;
- When DMA requests are served, the input data are temporarily stored in the FIFO buffer. Data extraction from the FIFO and/or processing is triggered by the receive interrupt. One problem with this method is message end detection. This can be performed by detecting an End-of-Frame (EOF) character or by detecting the pause at the end of the data block.

The following presents the implementation of the timeout to detect the 3.5-character length without transmission, end of message, or receive DMA timeout. If the receive DMA is used, the end of the transfer cannot be detected unless the number of bytes to be transferred is known in advance. As a result, a DMA timeout should be implemented when no more data are received. For this, two methods are presented in [37], namely:

- Connecting USART_RX to the capture input of a timer;
- Using a system clock and interrupt reception from USART.

**Figure 6:**
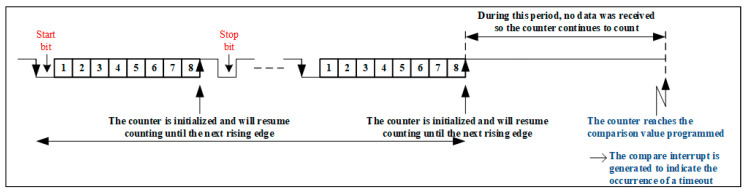
Method 1—Connecting USART_RX to the capture input of a timer.

For the first method (Figure 6), i.e., connecting the USART_RX to the capture input of a timer, the method is to use a timer in slave reset mode, whose counter is reset in response to the rising edge of a signal connected to the receive pin of the USART (USART_RX). On each rising edge on the receive input pin, the counter is reset. By programming the comparison output value with the desired timeout, when no more data are transmitted, the counter continues its operation until the comparison value for the user-defined timeout is reached. 

**Figure 7:**
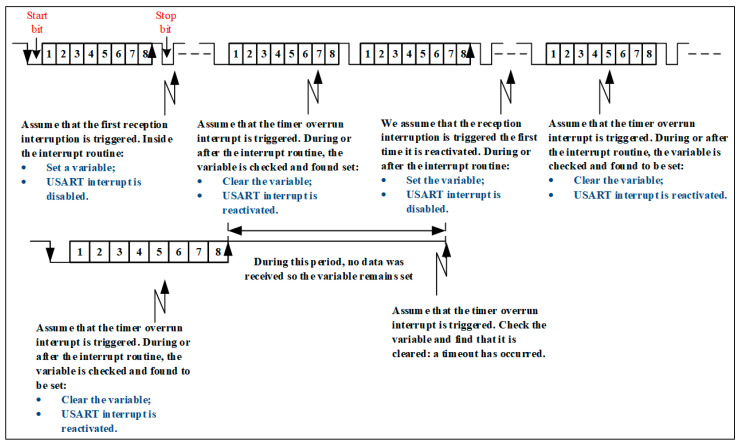
Method 2—Using a system clock and interrupt reception from USART.

Consequently, the timer generates a comparison interrupt (already activated) which informs the application about the occurrence of the timeout. Figure 6 illustrates this first method. As for method 2 (Figure 7), a system clock and the USART receive interrupt of the STM32F4 microcontroller are used. This method is used to detect a timeout in the 1 ÷ 2 period range of the system clock. Figure 7 illustrates this method, with the mechanism working as follows:

- Validate the USART receive interrupt and DMA requests;
- Validate system clock overflow interrupt;
- When the USART receive interrupt is triggered, set a variable and disable the receive interrupt. This ensures that at least one receive interrupt has occurred;
- When the overrun interrupt is triggered at the timer, the value of the variable is checked, and the following actions are performed:
  a. If it is set, it means that no timeout has occurred. The variable must be deleted and the receive interrupts must be reactivated.
  b. If cleared, this means that a timeout has occurred, i.e., no reception interrupts have occurred during the programmed time.

**Figure 8:**
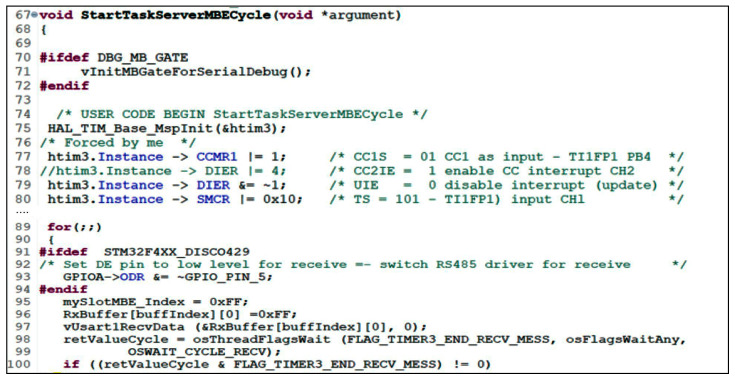
Start timer 3 for retrigger mode and signal to myTaskServerMBEcycle thread.

**Figure 9:**
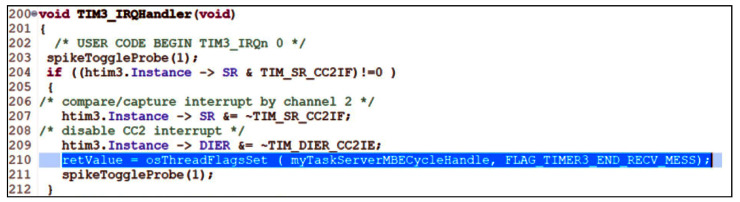
Interrupt handling routine generated by timer 3 signaling end of ModbusE received message.

Regarding the solution chosen for the MBU_i server regarding the DMA timeout, the first solution was to use a timer in the RESET slave mode. It works as a resettable monostable, the external signal used being the receive signal, more precisely its rising edge (pin PA10 was connected to PB4). This edge resets the timer counter. If there are no edges, the timer increments up to a comparison value at which, if reached, an interrupt is generated signaling the end of reception. The initialization sequence of this operation is shown in Figure 8. Figure 9 illustrates the interrupt handling routine generated by the timer when the receive line goes into IDLE. 

The interrupt handling routine clears the compare interrupt and cancels the interrupt given by timer 3. Using the RTOS function, osThreadFlagSet signals the task myTaskServerMBEcycle that a new message has been received (Figure 8). Another aspect presented is the signaling of the beginning of the message. Thus, it is important to detect when the reception of a message starts. The USART1 circuit provides an IDLE interrupt, which signals the first falling edge after the duration of an IDLE character (10, 11 bits per 1). The routine for handling this interrupt (USART interrupt) will allow clearing the interrupt given by the IDLE status, deactivating it, and starting timer 3 to handle the end of the message. The vUsart1RecvData function is intended to prepare USART1 for the message read operation. This function performs the following:

- RS485 driver receive switch (slightly redundant);
- Deinitialize and reinitialize DMA2-stream 2 channel for receive and fetch and program DMA with counter for number of bytes to be transferred (maximum receive buffer length (2 buffer-e)), memory address of receive buffer used, address of DR receive register in USART1, and enable DMA channel, i.e., transmission complete (TC) interrupt flag given by counter termination;
- Activate USART1 for DMA reception;
- Clearing the IDLE interrupt at USART1 if any;
- Activate the IDLE interrupt to trace the start of the message at USART1.

The vUsart1SendData function is responsible for preparing USART1 for the message write operation. This function performs the following actions:

- Switching the RS485 driver to transmit;
- Programming DMA2-stream 7 for transmission, retrieving and programming the DMA with the counter for the number of bytes to be transferred for a maximum receive buffer length of 2 e buffers, the memory address of the transmit buffer used, and the address of the DR transmit register in USART1, and activating the DMA channel or the TC interrupt given by the counter termination.

**Figure 10:**
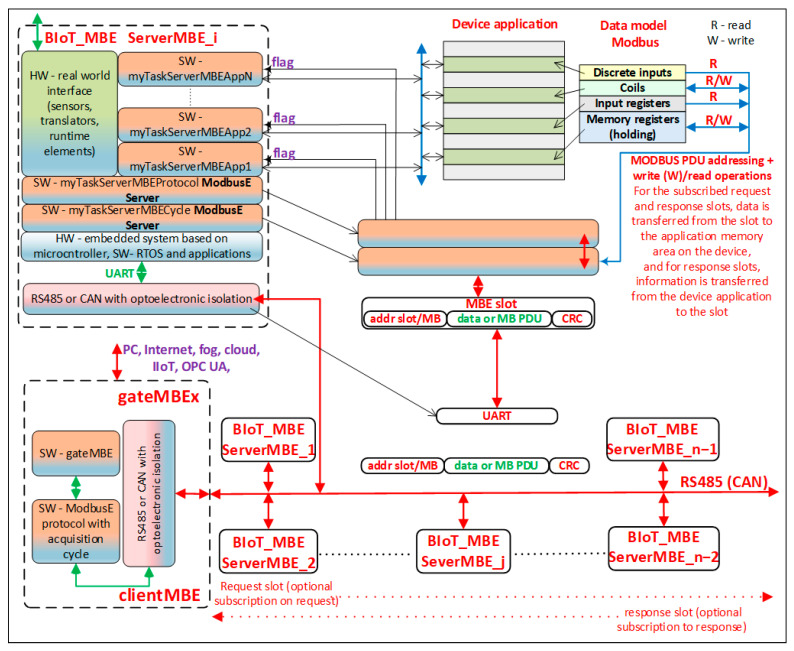
ModbusE acquisition slot and ServerMBE_i device.

The slot is a basic message, both as a request and as a response, and is an integral part of an acquisition cycle. The slot has three parts, namely slot address or Modbus address, data, and CRC (Figure 10). The characteristics of ModbusE slots are as follows:

- Slots are composed of 0 or more sections, and all slots in the 0 and 1 range have both request and response;
- The length of a slot may be 3 and a maximum of 256 bytes, and the user slot address may be between 2 and 127 (MAX_SLOTS). Request or response slots may not have a data field;
- The addresses in the acquisition cycle are sequential addresses between 0 (0 and 1 are special request type slots) and maxim-1 slots in the acquisition cycle (0 … MAX_SLOTS_IN_LOOP-1);
- If more slots than MAX_SLOTS_IN_LOOP are defined in the device, slots outside the acquisition cycle (OUT_OF_CYCLE) may be used by indirection of slots in the acquisition cycle (IN_CYCLE);
- The slot address field may also be a Modbus address, and the data field may be a Modbus PDU (Protocol Data Unit);
- A slot to which the address is a Modbus device address is regarded as a Modbus APU (Application Data Unit);
- A request slot always requires a response slot (except for slots 0 and 1) and is only launched by the client;
- A request slot can be sent less frequently by defining a number of idle cycles for it. If this number, for example, is 5, then the request will be sent once every 5 slots;
- The last slot in the cycle can be a ModbusE slot with classic Modbus commands, usually the last slot in the cycle. Only one device responds on this slot, or none if it is faulty or outside the Modbus network;
- Request-type slots perform only write operations, based on the configuration of that slot, and response-type slots perform read operations to the device selected to issue the response or write operations to another device that has subscribed to the response.

For the device sending the request–response, the length of the request must be different from the length of the response slot. A server station can subscribe to both a request message and a reply message. If these two messages have the same address, the server station cannot distinguish whether it is the request or the reply. The length of the message is a parameter that can differentiate a request from a response. For this, the request length must be different from the reply length. Server devices do not have an FSM to know that they have received a request or a reply. With different message lengths and message types, it is easy to differentiate. As can be seen in the block diagram shown in Figure 10, the USART is programmed on receive with DMA to retrieve messages from the RS485 bus. 

Thus, the end of the message, signaled with a timer-based mechanism in slave RESET mode, allows signaling to the myTaskServerMBEcycle task, implemented with the name StartTaskServerMBEcycle, the reception of a message. This task checks if the message has the correct CRC, reads the message length from the DMA, and then checks if the slot address is part of the slots parsed by the device. The number of these slots, as well as slots 0, 1, and the slot dedicated to asynchronous Modbus messages with an acquisition cycle, is signaled to the myTaskServerMBEProtocol task, implemented as StartTaskServerMBEProtocol. This task will handle the new values in collaboration with the other tasks cooperating on updates to the device application memory using the Modbus data and addressing model. If the slot address does not match the device’s Modbus address for the request and response subscription slots, the myTaskServerMBEcycle task moves the data from the slot to the application memory organized by Modbus data and addressing model. This operation is performed with the function “void vexecuteRecvSlotWriteRegs(uint8_t SlotIndex)” given the Modbus write functions 5, 6, 15, 16, 22, 23 based on the information in the MY_SLO_PARAM slot structure. When the current device sends the response on the bus, if the slot address does not match the device’s ModbusE address for the corresponding response slots, the myTaskServerMBEcycle task moves the data from the application memory organized by Modbus data and addressing model into the slot with the function “void vexecuteRecvSlotWriteRegs(uint8_t SlotIndex)” given the Modbus read functions 1, 2, 3, 4, 7, 11, 23 based on the information in the MY_SLO_PARAM slot structure. For slots with Modbus APDU, i.e., when the slot address matches the Modbus address of the device, the myTaskServerMBEcycle task moves the data and responds if necessary according to the Modbus specifications for functions 1, 2, 3, 4, 5, 6, 7, 11, 15, 16, 22, 23, directly using the information in the request with the function “void vModbusExecuteFunctions(void)”. As the highest priority task, it will perform atomic read/write operations. Thus, the myTaskServerMBEProtocol task and application tasks will also need to implement atomic access to this application memory area organized by Modbus data and addressing model, involving a critical section.

The following is the pseudocode of the myTaskServerMBECycle task implemented by the StartTaskServerMBECycle function. In the case of Algorithm 1, the execution time of a message is shorter, and the processing is taken over by the StartTaskServerMBEProtocol task. Thus, the StartTaskServerMBECycle task can switch to receive and accept a new message without the previous one being processed. In this case, the response time of the StartTaskServerMBEProtocol can be increased, but this solution may undesirably affect more slots that could be executed faster. Also, one can synchronize the StartTaskServerMBEProtocol and StartTaskServerMBECycle tasks using a flag or an event, or work alternatively with two receive buffers. Thus, it will also be necessary to implement a buffer at the level of the StartTaskServerMBEProtocol task in order to release the receive buffers urgently. In the case of implementing other algorithms, it can move directly into and out of the register file, register entries, coils, and discrete entries. 

Algorithm 1, proposed and implemented for the StartTaskServerMBECycle task, although having more lines of pseudocode than other implementations, is shorter in execution time because it passes the proper tasks of reading/writing slot data, executing ModbusE messages, and parsing the slot configuration using function 100 to the StartTaskServerMBEProtocol task. As for the proposed algorithm, if the length of the received message is equal to the length in the associated slotIndex structure and the message is a request to this server, then a request message has been received to which the server must respond, and the following steps are performed:

- Move the message from the current receive buffer to the read buffer of the myTaskServerMBEProtocolHandle task;
- Signals the MBE application task (myTaskServerMBEProtocolHandle) of this situation (RTOS function osThreadFlagsSet, event FLAG_SLOT_REPLAY_TO_SEND);
- Wait (RTOS function osThreadFlagsWait) for a limited time (OSWAIT_CYCLE_RECV) for the FLAG_APP_RESPONSE event which signals that the MBE application task (myTaskServerMBEProtocolHandle) has prepared the response in the transmit buffer, and it shall be sent over RS485;
- If the function returns an error (TEST_FLAGS_ERRORS), then it flags the error, increments the error counters, and returns to the infinite loop of the StartTaskServerMBECycle task.

If the function returns the FLAG_APP_RESPONSE event, then it initializes the already prepared response transmit state (vUsart1SendData function) and waits (osThreadFlagsWait RTOS function) for a limited time (OSWAIT_CYCLE_SEND) for the FLAG_UART_END_SEND_MESS event which signals that the message has been sent, this event being generated by the TC flag USART1 interrupt routine. If the wait function returns an error (TEST_FLAGS_ERRORS), then it signals an error, increments the error counters, and returns to the infinite loop of the StartTaskServerMBECycle task. If the wait function receives the FLAG_APP_RESPONSE event, then it increments the sent message counter and increments the receive buffer index, keeping the rest of the division at two (only two receive buffers).
**Algorithm 1.** StartTaskServerMBECycle task implementation logic for serverMBE stations1.StartTaskServerMBECycle2.Initialization3.**Infinite loop task:**4.  Switch the RS485 driver to receive (PA5 = 0)5.  Loop initializations (index and clear the CRC)6.  Initialize reception status (vUsart1RecvData function)7.  Waits (RTOS function osThreadFlagsWait) for a limited time (OSWAIT_CYCLE_RECV) for the FLAG_TIMER3_END_RECV_MESS event that signals the end of the reception of a message (set by timer interrupt 3)8.**  If** the FLAG_TIMER3_END_RECV_MESS event has arrived **Then**9.    Get the number of characters received in the current buffer10.    **If** the number of characters received is greater than 256 **Then**11.      Signals error, initializes receive buffer index12.      **GO TO** Infinite loop task13.    Increases the number of messages received (debugging, performance, etc.)14.    Get slot number15.    Calculate the received message CRC16.    **If** the CRC is incorrect **Then**17.      Increases the number of messages received in error (debugging, performance, etc.)18.      Initialize receive buffer index19.      **GO TO** Infinite loop task20.    Increases the number of messages received with good CRC (debugging, performance, etc.)21.    **If** slot = 0 **Then**22.      Signal the MBE application task (myTaskServerMBEProtocolHandle) of this situation (RTOS function osThreadFlagsSet, event FLAG_SLOT0_RECEIVED)23.      Loop initializations (index, CRC reset and slot = 0xFF)24.      **GO TO** Infinite loop task25.    If slot = 1 Then26.      Signal the MBE application task (myTaskServerMBEProtocolHandle) of this situation (RTOS function osThreadFlagsSet, event FLAG_SLOT1_RECEIVED)27.      Loop initializations (index, CRC reset and slot = 0xFF)28.      **GO TO** Infinite loop task29.    Search an array of slot based structures for a (slotIndex = 0xFF with which to further identify the associated structure for the slot30.    **If** slotIndex = 0xFF **Then** no slot was found used by this serverMBE31.      Loop initializations (index and slot = 0xFF)32.      **GO TO** Infinite loop task33.    **If** the length of the received message is equal to the length in the associated slotIndex structure, and the message is a reply from another server, **Then** a reply message has been received to which the server has subscribed34.    Move the message from the current receive buffer to the read buffer of the myTaskServerMBEProtocolHandle task35.    Signal the MBE application task (myTaskServerMBEProtocolHandle) of this situation (RTOS function osThreadFlagsSet, event FLAG_SLOT_REPLAY_RECEIVED)36.      Loop initializations (index, CRC reset and slot = 0xFF)37.      **GO TO** Infinite loop task38.    **If** the length of the received message is equal to the length in the associated slotIndex structure, and the message is a request to another server **Then** a request message has been received to which the server has subscribed39.    Move the message from the current receive buffer to the read buffer of the myTaskServerMBEProtocolHandle task40.    Signal the MBE application task (myTaskServerMBEProtocolHandle) of this situation (RTOS function osThreadFlagsSet, event FLAG_SLOT_REQUEST_RECEIVED)41.    Loop initializations (index, CRC reset, and slot = 0xFF)42.    **GO TO** Infinite loop task43.  **End If**44.**End** Infinite loop task45.**End** StartTaskServerMBECycle function

Otherwise, the slot, although it exists, has none of the tested states (subscribe, reply, or request, or must reply to client request) performing loop initializations, such as index initialization, CRC, cancel, and slot = 0xFF, and returns to the infinite loop of the StartTaskServerMBECycle task. If an error was returned for the receive message event, then an error must be flagged and the error counter incremented, respectively. The disadvantage of the proposed algorithm for implementing the StartTaskServerMBECycle task is that it relatively has many lines of pseudocode and it transfers the actual tasks of reading/writing data, executing Modbus messages and configuration to the StartTaskServerMBEProtocol task. Thus, the algorithm can be improved to allow a better evaluation of the message processing time relative to the total execution time of the microcontroller [38].

### 4.3. Experimental Results

**Figure 11:**
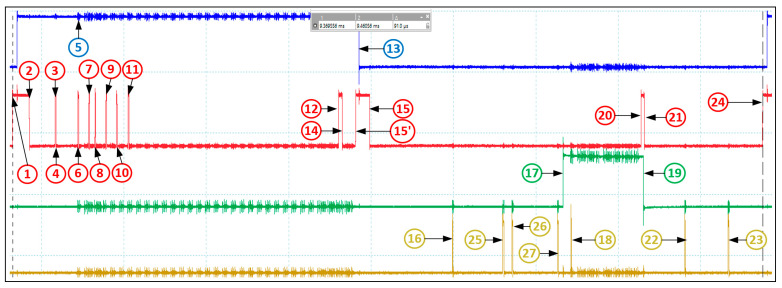
Important times within a ModbusE based tSi slot (Table 2).

**Figure 12:**
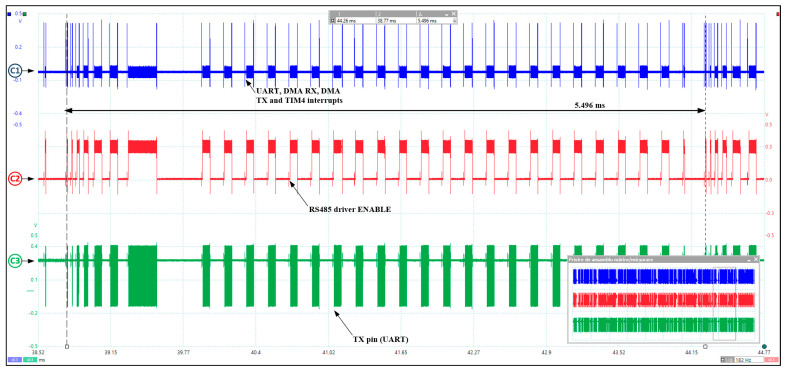
ModbusE acquisition cycle structure (Cursor C1 (blue)—spy spikes for UART TC, DMA RX TC, DMA TX TC, and TIM4 interrupts; Cursor C2 (red)—RS485 driver command; Cursor C3 (green)—UART TX output).

The important slot-level timings for ModbusE implementation are shown in Figure 11 and Table 2. These results were obtained using the STM32F4 development kit, with the Cortex-M4 CPU running at 168 MHz, 1 MB flash per chip, 196 KB SRAM, Cortex debug, and ETM Trace. For compatibility, the 15′ position has been additionally introduced. For the same reason, the additional numbers 25 to 27 for the server were inserted after the number 24, although temporarily they are before 24. Table 2 shows the measured acquisition cycle slot times for the ModbusE implementation, considering tintDMArx equal to 0 in slot 0 and for slots 1 to 29 tintDMArx being 0.338 μs. Also, tintT4i is in the range 1.992 ÷ 2.142 μs. As an observation, slots 0, 1, 2, and 3 also include the DMAtx + USART interrupt in tthd, the notation “+int” indicating that the thread is interrupted by the dmatx and usart interrupts, with “−int” indicating that the thread is not interrupted. The structure of the acquisition cycle is shown in Figure 12, the period measured in the oscilloscope capture is 5.496 ms. The time period tmCRC is the CRC processing time for the message received in the previous slot. The time period for slot i denoted by tSi measures the software processing time at the server. Added to this is the hardware delay time at the server, noted by thwSi, the times tcommSi, tcommMi, and tcommi are required to issue server and client characters via the response and request message, and tmswitchi is the time required to switch the highest priority task in the mbeThreadCycleRTU system.

The time period labeled tmpsnsli defines the time to prepare to issue the command for the new slot i, and tmfosli is the time to complete the operations related to the old slot corresponding to the client.

As part of the research activity for the implementation of the Modbus RTU VirtualComm gateway server with USB connection and ModbusE client with half-duplex, RS485 serial connection, the application project was realized using the STM32 IDE CUBE development environment and the STM32F429I-DISCO development kit. As a derivative contribution, the StartTaskCycle task was implemented, which incorporates the ModbusE concept on the gateway MBE client side. An example for a 30-slot acquisition cycle is also made by implementing a primary test by measuring the oscilloscope queries for slots 0 to 29. This software module is useful for testing the MBE server, including the RTU server. In this paper, a summary of the most important information to be considered for the implementation of the ModbusE server stations connected to the BIoT gateway was summarized, adapted for the STM32F429I-DISCO kit, and tested on the myTaskUsbServer task for ModbusRTU protocol as a server for a client placed on a PC using VirtualComm implemented on USB. So, for compatibility with a possible Modbus TCP-IP server, the myTaskMbeGate task was ported, adapted, and tested. Then, the myTaskDispatcheS task, the gateway dispatcher that provides the link between the MosbusE server and the Modbus RTU client, was ported, adapted for the STM32F4 kit, and tested, followed by brief tests using the ModbusPoll utility.

The contributions of the research carried out in this paper are as follows:

- Requirements and specifications for serverMBE stations have been proposed;
- The basic architecture of the serverMBE_i application has been defined;
- Implementation issues and solutions have been addressed, such as the management of the Driver Enable signal for the RS485 line driver, the FIFO buffers, and the DMA, the implementation of the timeout to detect the period of 3.5 characters without transmission representing the end of a message, and the signaling of the beginning of the reception of a message;
- The functions vUsart1RecvData, vUsart1SendData, and u16MODBUS_CRC16_table16 have been implemented;
- The pseudocode for the StartServerMBECycle task has been presented and the corresponding code has been written;
- The function 100 (Modbus function parser) extension of ModbusE that allows slots to be defined using Modbus function parameters stored in a structure that allows them not to be sent over the network, has been proposed and implemented, resulting in a major optimization of data channel throughput. Requirements of serverMBE stations have been outlined.

## 5. Conclusions

BIoT provides Internet connection and the remote management of mobile devices through various sensors and actuators that measure building conditions. In this way, the operation of SB appliances can also be monitored. Cloud computing provides computing power and storage space to develop, maintain, and run services at home anywhere, anytime. Based on the requirements of serverMBE stations in a ModbusE-based BIoT communications architecture, it is possible to clearly specify the aspects of request–response messages that are transmitted on the physical ModbusMBE layer. Thus, the request is always initiated by a client device, and only one client is active on a ModbusMBE bus at a time. However, a command can be provided to allow a token to move between multiple clients. From the device’s point of view, the request is always a request to which the device has subscribed, so it can include slot addresses 2 … 128 but also classic Modbus addresses such as 128 … 247. The response, if any, excluding slot 0 and optionally address 1, is issued by a server station that owns that slot or Modbus address. Another station may subscribe to this response if its response slot configuration includes that station’s respective response slot.

The results obtained by the authors in accordance with the ModbusE protocol refer to the fact that only data and checksum are transmitted in a slot, thus increasing the bandwidth utilization of the communication channel, since headers are no longer transmitted. The data meaning is defined either by configuration or by classical Modbus commands in system initialization. Based on ModbusE, data acquisition systems can be developed as modules for digital inputs/outputs and analog inputs such as voltage, current, and analog outputs. These modules are supervised by BSG, which has a defined acquisition cycle. Thus, a time stamp can be added corresponding to the acquisition time of the modules or the time when the data are read from the BSG. These modules, together with the BSG, can be used in any industry sector or BIoT where monitoring of data from different sensors is required. Connection of the BSG to a PC or other industrial computer is made by the Modbus TCP/IP protocol.

As future research directions, an improved algorithm for the myTaskServerMBECycle task will be developed. For this algorithm, the synchronization between these two tasks may entail the use of mutexes that can lead to task switching and priority inversions that extend the execution time of operations on the transmit/receive buffers. As a result, per total receive/transmit communication operations and processing of received or transmit information, the algorithm is likely to become shorter and more reliable. So, in what follows, a comparison of the advantages and disadvantages of each algorithm will be made.

## 6. Patents

The smart switch embedded system device including the Modbus Extension server is based on the patent application (OSIM CBI A/00224, 2023) filed by the authors for BIoT systems.

## Figures and Tables

**Table 1:** The Modbus absolute address ranges (984 scheme) and data model.

Table Name and Operations Allowed	Data Addreasess and Type	Modbus Codes	Coil/Register Numbers
Discrete outputs (“coils”), read (R)/write (W)	0000 ÷ 270E/1 bit	01 (R), 05 (W single), 15 (W multiple)	00001 ÷ 09999
Discrete inputs (“contacts”), R	0000 ÷ 270E/1 bit	02 (R)	10001 ÷ 09999
Analog input registers, R	0000 ÷ 270E/16-bit size	04 (R)	30001 ÷ 39999
“Holding” registers, R/W	0000 ÷ 270E/16-bit size	03 (R), 06 (W single), 16 (W multiple)	40001 ÷ 49999

**Table 2:** Measured times for acquisition cycle slots based on ModbusE implementation (in μs).

Slot	tmCRC	tmintDMAtx + tmintUSART	tmcommi	tmswitchi	tmpsnsli	tmfosli	tthd + dmatx + usart	tSi	tCYA
0 (3/0)	0	0.451 + 0.846	3.721	2.998	2.847	2.565	8.936	41.04	+int
1 (4/0)	0	0.451 + 0.846	4.792	3.101	2.678	2.058	8.908	42	+int
2 (16/4)	0	0.507 + 1.748	17.37	3.101	2.65	1.41	6.953	64.05	−int
3 (32/8)	1.24	0.451 + 1.748	34.05	3.101	2.65	3.326	9.021	91	−int
4 (64/16)	2.03	0.451 + 1.691	67.54	3.157	2.706	4.059	9.585	144.0	−int
5 (64/32)	3.383	0.451 + 1.804	67.66	3.157	2.819	5.525	11.16	160.9	−int
6 (256/256)	5.788	0.507 + 1.734	267.4	3.165	2.781	8.156	13.53	661.2	−int
7 (64/64)	43.09	0.532 + 1.766	67.66	3.061	2.802	43.35	50.87	193.9	−int
8–28 (64/64)	11.57	0.426 + 1.777	67.60	3.142	2.702	13.33	19.06	193.9	−int
29 (slot 1 indirection)		2.555	4.879	3.177	2.741	14.88 +int	20.65 +int	194	+int
									Total 5.673 μs

## Data Availability

Data are contained within the article.
